# Introducing the global antimicrobial stewardship partnership hub (GASPH): creating conditions for successful global partnership collaboration

**DOI:** 10.1093/jacamr/dlac115

**Published:** 2022-11-08

**Authors:** Jacqueline Sneddon, Tracey Guise, David Jenkins, Mirfin Mpundu, Maarten Van Dongen, Jeroen Schouten, Jonghong Xiao, Gloria Cordoba, Dilip Nathwani

**Affiliations:** British Society for Antimicrobial Chemotherapy, Birmingham, UK; British Society for Antimicrobial Chemotherapy, Birmingham, UK; British Society for Antimicrobial Chemotherapy, Birmingham, UK; Department of Microbiology, University Hospitals of Leicester NHS Trust, Leicester, UK; ReAct Africa, Lusaka, Zambia; AMR Insights, Amsterdam, The Netherlands; Department of Intensive Care and Radboudumc Center for Infectious Diseases, European Society of Clinical Microbiology and Infectious Diseases (ESCMID) Study Group on Antimicrobial Stewardship (ESGAP), Nijmegen, The Netherlands; School of Medicine, Zhejiang University, Hangzhou, China; International Centre for Antimicrobial Resistance Solutions, Copenhagen, Denmark; British Society for Antimicrobial Chemotherapy, Birmingham, UK

## Abstract

In 2015, 196 countries formally committed to a Global Action Plan to address antimicrobial resistance (AMR). However, translating policy into practice is not happening at pace and the recent Global Research on AntiMicrobial resistance (GRAM) Project report confirms the burden of AMR is increasing. Despite progress in establishing surveillance data and investment in new antimicrobials, education and training including use of behavioural science approaches to change practice is lagging. To contribute to addressing this, we have invited organizations to join us as founding members of the Global Antimicrobial Stewardship Partnership Hub (GASPH) (https://global-asp-hub.com/). We will work together to share education resources and foster collaboration to meet the needs of learners and of partner organizations working on tackling AMR. Membership is open to all—professional societies, academic institutes, nongovernmental organizations/civil society, philanthropists and commercial partners interested in supporting a multi-stakeholder global antimicrobial stewardship (AMS) education platform and network.

Despite the commitment of 196 countries in 2015 to a Global Action Plan^1^ to address AMR, the translation of policy into clinical practice is not progressing at the pace needed to tackle the slow burning pandemic of AMR.^2^ The report on the Global Research on AntiMicrobial resistance (GRAM) Project study^3^ published in January 2022 provides the first comprehensive assessment of the global burden of AMR together with an evaluation of the availability of data. The report confirms AMR as a leading cause of death globally, with the highest burdens in low-resource settings. To support location-specific policy decisions, we need to understand the burden of AMR and the leading pathogen–drug combinations contributing to it. This is essential to inform infection prevention and control programmes, access essential antibiotics, and for the research and development of new vaccines and antibiotics. This report confirms the serious data gaps in many low-income settings and the need to expand microbiology laboratory capacity and data collection systems. In addition, from global, national and local perspectives, education and training on AMS are needed to accelerate change in how we use antimicrobials to address AMR. We need to enable better understanding of how policy supports practice and vice versa, to assess the impact of education initiatives and, given the limited funding available, to avoid a duplication of effort in developing, delivering and sharing resources. This may help address the lack of action on the implementation of AMR National Action Plans worldwide.^4^

Low- and middle-income countries (LMICs) and high-income countries would benefit from enhanced opportunities to work collaboratively. It is important that the global AMS community supports both LMICs and high-income countries to lead the development and implementation of education solutions to the problem of AMR within their economic setting with reciprocal benefits for all. We require a better understanding of how to establish cohesive, structured education and training provision for AMS programmes across all health economies, and how the impact of such programmes can be measured. There is also a need to address the inconsistent and fragmented provision of education and training, as demonstrated by a recent mixed methods study in India that concluded a formal framework is needed to support and legislate for AMS education and training.^5^ This requirement is undoubtedly the same across continents as echoed in the literature in recent years. A call for global collaboration was published in *The Lancet* headlining that, ‘united we succeed, divided we might fall’ in 2017^6^ and again in 2020 when a coalition of professional society groups similarly advocated the importance of education collaboration with a focus on the implementation challenges for AMS in LMICs.^7^

Ultimately, education coupled with knowledge transfer to support behaviour change is the common implementation pathway for AMS supporting effective research, innovation, new technologies, data science and new therapies into everyday clinical practice. Despite inadequate new or additional funding for education, such as exists for research and development or surveillance, there is much success to celebrate and to build on. Several organizations, including universities, learned societies, pharmaceutical and diagnostics companies, are working singularly or collaboratively to address the knowledge exchange, education and training needed to effect change.^8^ There are a growing number of examples of government supported initiatives aimed at bridging the divide, such as the Commonwealth Partnerships for the Antimicrobial Stewardship programme,^9^ awarded by the Tropical Health Education Trust and Commonwealth Pharmacist Association and funded by the UK Aid Fleming Fund. There are growing repositories of e-learning courses and online training, the technology for which has been one positive outcome of the COVID-19 pandemic detailed in a recent literature review on its impact on teaching and learning.^10^

The world has awakened to the liberty and freedoms afforded by recent advancements in technology that offer opportunities to connect with each other across the globe, to deliver education, share experiences and work together virtually in ways unimaginable only a decade ago.^11,12^ Against this landscape, and to address the identified education needs we are excited to have launched the GASPH (https://global-asp-hub.com/), hosted by the British Society for Antimicrobial Chemotherapy (BSAC), with the aspiration of establishing a truly cooperative global community dedicated to addressing the challenges of AMR through shared education, training and tacit learning. The mission of GASPH is to accelerate the PACE of action on AMR through Partnership, Advocacy, Commerce and investment, and Education. Representatives from over 20 partner organizations joined the GASPH launch webinar on 30th May 2022 and there was much enthusiasm for working together to support shared learning. The provision of an open access e-learning, knowledge sharing platform will amplify, promote and enhance the work undertaken by a range of stakeholders now and in the future. Regular virtual meetings will support sharing of outputs from collaboration and stimulate discussion of key issues, exploration of further areas for collaborative working and development of new relationships. The GASPH platform will be supported via the BSAC secretariat with governance provided via a multi-professional, WHO-categorized regionally representative Expert Steering Committee and partner organizations collaborating via Global Advisory Board webinar meetings three times per year. Partners organizations will be multi- professional ranging from professional societies, NGOs and others. Their primary focus will be to actively promote or champion sharing and curation of digital learning within their own and the broader communities and create a global knowledge network focused on education and ‘real world’ implementation of AMS and broader AMR interventions. This focus on open access to digital training resources, thereby reducing costs and fostering greater buy-in from professional societies will help to overcome barriers identified^10^ to participating in AMS training. The combination of online learning, with face-to-face training where required and remote expert coaching may prove to be most useful to develop and embed AMS practice. The key benefit of joining GASPH is shared learning with free membership for partner organizations wishing to share resources including good practice examples and for individuals seeking access to education and training. To date, our e-learning repository has 765 resources from 38 countries across six continents (most are peer-reviewed) and our training hub includes 32 online courses. We acknowledge the potential challenges of digital resources in some settings and the language requirements of learners. Most resources are in English, but we are exploring how to increase access to content in other languages.

We hope you will join us on this journey to achieve our ambition of an open access education utopia that will support our mission to use shared knowledge and experience to slow the AMR pandemic.

## References

[1] World Health Organization . Global action plan on antimicrobial resistance. https://www.who.int/publications/i/item/9789241509763; 2015.

[2] Weldon I , Hoffman SJ. Bridging the commitment-compliance gap in global health politics: lessons from international relations for the global action plan on antimicrobial resistance. Glob Public Health 2021; 16: 60–74. 10.1080/17441692.2020.1788623 32623966

[3] Murray CJL , Ikuta KS, Sharara F et al Global burden of bacterial antimicrobial resistance in 2019: a systematic analysis. Lancet 2022; 399(10325): 629–55. 10.1016/S0140-6736(21)02724-0 35065702 PMC8841637

[4] Charani E , Mendelson M, Ahmad R, et al National action plans for antimicrobial resistance: a framework for operationalising existing plans and developing a research strategy to address gaps and opportunities. *ECCMID Conference,* 2022. Abstract 02857.

[5] Singh S , Charani E, Wattal C et al The state of education and training for antimicrobial stewardship programs in Indian hospitals—a qualitative and quantitative assessment. Antibiotics 2019; 8: 11. 10.3390/antibiotics8010011 PMC6466562 30704037

[6] Goff DA , Kullar R, Goldstein EJC et al A global call from five countries to collaborate in antibiotic stewardship: united we succeed, divided we might fail. Lancet Infect Dis 2017; 17: e56–63. 10.1016/S1473-3099(16)30386-3 27866945

[7] Tattevin P , Hara GL, Toumi A, et al Advocacy for increased international efforts for antimicrobial stewardship actions in low-and middle-income countries on behalf of alliance for the prudent use of antimicrobials (APUA), under the auspices of the International Society of Antimicrobial Chemotherapy (ISAC). Front Med. 2020; 7: 503. 10.3389/fmed.2020.00503 PMC7479847 32984380

[8] Infection Learning Hub, British Society for Antimicrobial Chemotherapy. https://infectionlearninghub.co.uk/.

[9] Tropical Health Education Trust, Commonwealth Partnerships for Antimicrobial Stewardship. https://www.thet.org/our-work/grants/cwpams/.

[10] Pokhrel S , Chhetri R. A literature review on impact of COVID-19 pandemic on teaching and learning. Higher Education for the Future 2021; 8: 133–41. 10.1177/2347631120983481

[11] Weier N , Nathwani D, Thursky K et al An international inventory of antimicrobial stewardship (AMS) training programmes for AMS teams. J Antimicrob Chemother 2021; 76: 1633–40. 10.1093/jac/dkab053 33738498

[12] Wernli D , Jørgensen PS, Parmley EJ et al Evidence for action: a one health learning platform on interventions to tackle antimicrobial resistance. Lancet Infect Dis 2020; 20: e307–11. 10.1016/S1473-3099(20)30392-3 32853549 PMC7444982

